# The Freedom of the Profession

**Published:** 1904-01-23

**Authors:** 


### THE FREEDOM OF THE PROFESSION.

AMONG the many popular misconceptions regarding the medical profession there exists a widespread suspicion that its followers are subject both in opinion and practice to certain definite rules. These, it is supposed, constitute an official orthodoxy and departure from them leads either to statutory penalties or to the adoption of a professional boycott. The suggested justification for this view is found in the disciplinary action of the General Medical Council and of the licensing corporations, and also in the refusal of professional recognition to those who ostentatiously defy certain generally recognised rules of conduct. It were perhaps hopeless to expect any rapid modification of this unjust and prejudicial theory. The history of a remote past may be quoted in its support, and malicious or ignorant misrepresentation may turn the most innocent practices to its purpose. But it is well for the profession to recognise its own freedom and to protest this as opportunity offers to those without its ranks. Of the fact itself there is of course no question. Neither subscription to a creed nor assent to a formula is among the demands made on those who aspire to enter the profession of medicine. Opinion from the outset is unfettered; it is also free to undergo development and modification as experience dictates. The contrary view, though it commands so much popular acceptance, is seen to be an absurdity to anyone acquainted with modern medical literature and teaching. Here are to be seen in sharp conflict directly antagonistic doctrines and opinions, and though rival professors may urge their respective views with vehemence and even with the more questionable devices of the controversialist there is never an appeal to the sanction of an imperative voice or a threat of the penalty of proscription. Whatever may have been the case in the past, the reign of orthodoxy, in medicine at all events, is at an end and every man "may think the thing he will." There is also equal freedom in practice. The truth in short is that the practitioner is not only free to select, but is morally bound to select, whatever he honestly believes is likely to be beneficial to his patient. For sincere opinion and honest action, even though mistaken, there is always room and welcome in the catholic atmosphere of medicine. The innovator may ever be sure of a hearing and also of disciples if he can support his position by facts and sound reasoning, and no new departure the result of *bonâ fide* observation and argument has need to fear the terrors of authoritative suppression and banishment. It is true that there are certain practices towards which the profession turns a severe and unyielding front. But these on examination will be found to involve a restriction of the liberty which it is the duty, not less than the pride, of the profession to claim. It is those who boast a special system as the exclusive repository of therapeutic truth, not the general body of the profession, who must be held guilty of intolerance and exclusion. Such, if true to their creed, bind their hands both now and for the future to a foregone conclusion and renounce the full liberty of thought and action which science equally with prudence enjoins. But even all this is possible without the risk of authoritative censure or professional ostracism. The teacher of an exclusive doctrine may advocate his theory in the usual organs of professional opinion and may put it into operation on all patients seeking his advice without any danger to himself or his professional position. However mistaken he may be believed to be no one will cry for his exclusion or degradation. It is, however, the case that some of those who claim a monopoly of truth are not content with the free arena of intra-professional discussion. They aspire for the sympathy and attention of those members of the public who have ever a ready ear for proclamations of peculiar personal ability and virtue, more especially when these are seasoned with the piquant suggestion of professional persecution. The adoption of distinctive names secures this end. Hence those who voluntarily affect such titles seek the judgment of a non-professional audience. The fact that this meets with professional disapproval is no contradiction of the claim of freedom in medical thought and practice. Rather it is the natural attitude to adopt to those who divest themselves of that freedom in circumstances which suggest that they are more anxious to announce their superior righteousness than to establish their doctrines in the judgment of their peers.

